# Supplementary material for: Drug repurposing for aging research using model organisms
Source: Aging Cell. 2017 Jun 16;16(5):1006–15. doi: 10.1111/acel.12626 (PMC5595691; doi:10.1111/acel.12626)
Supplement: Supplementary file 7 — Data S1 Zip‐Archive of all report cards. [file ACEL-16-1006-s007.zip › RC_G96.pdf]

G96

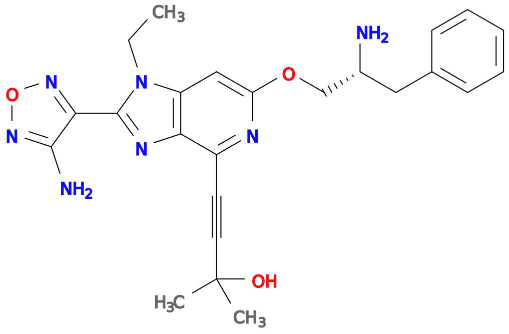

Database identifiers

ChEMBLCompound	CHEMBL1098938

Ranking

	Rank	Score
Drosophila	162/697	0.733
C. elegans	230/591	0.233

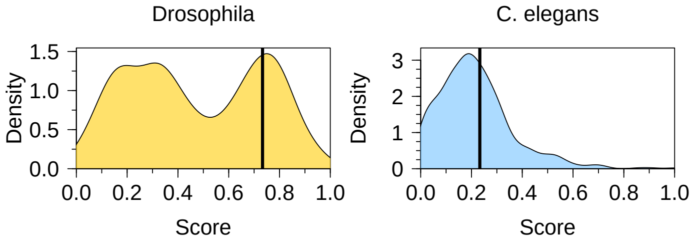

	Ageing implication	Domain conservation	Binding site conservation	Binding affinity	Bioavailability	Lipinski	Promiscuity	Purchasability	Drug approval	**Total**
Drosophila	1.0	0.952	0.923	0.928	(0.9)	0.0	-0.0	0.0	0.0	0.733
C. elegans	1.0	0.925	0.981	0.928	0.277	0.0	-0.0	0.0	0.0	0.233

Names

No synonyms found

Roles

ChEBI entry None has no roles

Status

Approved drug (according to ChEMBL)	No
Number of Rule of 5 violations	0
Binding affinity to original target in log units (RF-Score prediction)	7.55
Burns *C. elegans* bioavailability prediction	-1.03

Compound Target Characteristics

RAC-beta serine/threonine-protein kinase

Best gene implication in ageing for this target family came from gene Q9XTG7 annotated in UniProt release 2014_02. Annotation GO 8340 (determination of adult lifespan) was Inferred from Mutant Phenotype

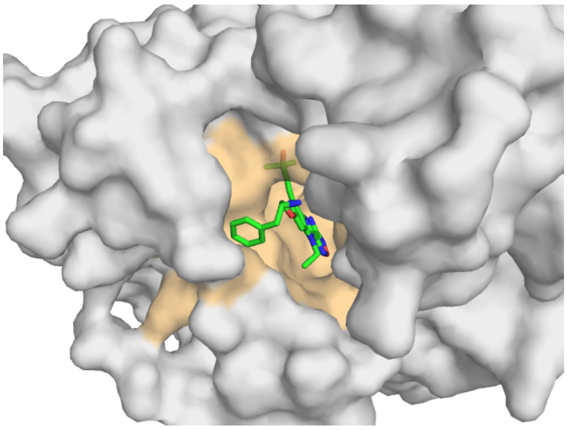

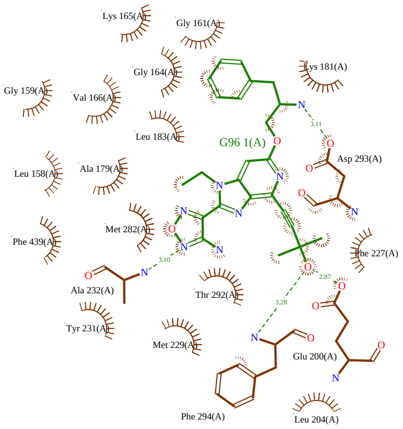

protein	amino acids contacts (binding site)																				
PDB:3e88:chainA:P31751	L	G	G	G	K	V	A	K	L	E	L	F	M	E	Y	A	M	T	D	F	F
tr:B3KP61:B3KP61_HUMAN	L	G	G	G	K	V	A	K	L	E	L	F	M	E	Y	A	M	T	D	F	F
tr:B7Z5R1:B7Z5R1_HUMAN	L	G	G	G	K	V	A	K	L	E	L	F	M	E	Y	A	M	T	D	F	F
tr:B4DG79:B4DG79_HUMAN	L	G	G	G	K	V	A	K	L	E	L	F	M	E	Y	A	M	T	D	F	F
tr:M0R0P9:M0R0P9_HUMAN	L	G	G	G	K	V	A	K	L	E	L	F	M	E	Y	A	M	T	D	F	F
sp:P31749:AKT1_HUMAN	L	G	G	G	K	V	A	K	L	E	L	F	M	E	Y	A	M	T	D	F	F
tr:B0LPE5:B0LPE5_HUMAN	L	G	G	G	K	V	A	K	L	E	L	F	M	E	Y	A	M	T	D	F	F
sp:P31751:AKT2_HUMAN	L	G	G	G	K	V	A	K	L	E	L	F	M	E	Y	A	M	T	D	F	F
sp:P47196:AKT1_RAT	L	G	G	G	K	V	A	K	L	E	L	F	M	E	Y	A	M	T	D	F	F
tr:Q3HSE5:Q3HSE5_RAT	L	G	G	G	K	V	A	K	L	E	L	F	M	E	Y	A	M	T	D	F	F
tr:Q8CE74:Q8CE74_MOUSE	L	G	G	G	K	V	A	K	L	E	L	F	M	E	Y	A	M	T	D	F	F
sp:Q60823:AKT2_MOUSE	L	G	G	G	K	V	A	K	L	E	L	F	M	E	Y	A	M	T	D	F	F
sp:P31750:AKT1_MOUSE	L	G	G	G	K	V	A	K	L	E	L	F	M	E	Y	A	M	T	D	F	F
tr:Q3TY95:Q3TY95_MOUSE	L	G	G	G	K	V	A	K	L	E	L	F	M	E	Y	A	M	T	D	F	F
tr:H1ZY64:H1ZY64_DROME	L	G	G	G	K	V	A	K	L	E	L	F	M	Q	Y	V	L	A	D	F	F
sp:Q8INB9:AKT1_DROME	L	G	G	G	K	V	A	K	L	E	L	F	M	Q	Y	V	L	A	D	F	F
tr:H1ZY63:H1ZY63_DROME	L	G	G	G	K	V	A	K	L	E	L	F	M	Q	Y	V	L	A	D	F	F
sp:Q9XTG7:AKT2_CAEEL	L	G	G	G	K	V	A	K	I	E	L	F	M	E	F	A	L	T	D	F	F
sp:P11792:SCH9_YEAST	L	G	G	G	Q	V	A	K	L	E	L	L	T	D	Y	M	L	C	D	F	F

protein	whole protein ident	simil	domain-based ident	simil	contact-based ident	simil
PDB:3e88:chainA:P31751	0.99	1.0	0.99	1.0	1.0	1.0
tr:B3KP61:B3KP61_HUMAN	0.79	0.79	1.0	1.0	1.0	1.0
tr:B7Z5R1:B7Z5R1_HUMAN	0.71	0.82	0.85	0.96	1.0	1.0
tr:B4DG79:B4DG79_HUMAN	0.87	0.87	1.0	1.0	1.0	1.0
tr:M0R0P9:M0R0P9_HUMAN	0.78	0.8	0.9	0.92	1.0	1.0
sp:P31749:AKT1_HUMAN	0.81	0.94	0.85	0.96	1.0	1.0
tr:B0LPE5:B0LPE5_HUMAN	0.81	0.94	0.85	0.96	1.0	1.0
sp:P31751:AKT2_HUMAN	1.0	1.0	1.0	1.0	1.0	1.0
sp:P47196:AKT1_RAT	0.81	0.94	0.85	0.96	1.0	1.0
tr:Q3HSE5:Q3HSE5_RAT	0.98	0.99	0.99	1.0	1.0	1.0
tr:Q8CE74:Q8CE74_MOUSE	0.72	0.73	0.98	1.0	1.0	1.0
sp:Q60823:AKT2_MOUSE	0.98	0.99	0.98	1.0	1.0	1.0
sp:P31750:AKT1_MOUSE	0.81	0.94	0.85	0.96	1.0	1.0
tr:Q3TY95:Q3TY95_MOUSE	0.98	0.99	0.98	1.0	1.0	1.0
tr:H1ZY64:H1ZY64_DROME	0.49	0.69	0.7	0.9	0.81	0.92
sp:Q8INB9:AKT1_DROME	0.49	0.69	0.7	0.9	0.81	0.92
tr:H1ZY63:H1ZY63_DROME	0.49	0.69	0.7	0.9	0.81	0.92
sp:Q9XTG7:AKT2_CAEEL	0.48	0.76	0.58	0.85	0.86	0.98
sp:P11792:SCH9_YEAST	0.2	0.42	0.43	0.8	0.67	0.79

Akt1 (FBgn0010379) associated phenotypes

RU486 conditional, body size defective, cell death defective, cell size defective, chemical resistant, chemical sensitive, circadian rhythm defective, decreased cell death, decreased cell number, decreased cell size, developmental rate defective, germline clone, heat sensitive, hyperplasia, increased cell death, increased cell size, lethal - all die before end of pupal stage, long lived, maternal effect, neuroanatomy defective, neurophysiology defective, nutrition conditional, oxidative stress response defective, partially lethal - majority die, planar polarity defective, polyphasic, short lived, size defective, small body, somatic clone, some die during pupal stage

(Information from FlyBase)

Akt1 (UniProt:Q8INB9) annotation

Function: Serine/threonine kinase involved in various developmental processes. During early embryogenesis, acts as a survival protein. During mid-embryogenesis, phosphorylates and activates trh, a transcription factor required for tracheal cell fate determination. Also regulates tracheal cell migration. Later in development, acts downstream of PI3K and Pk61C/PDK1 in the insulin receptor transduction pathway which regulates cell growth and organ size, by phosphorylating and antagonizing FOXO transcription factor. Controls follicle cell size during oogenesis. May also stimulate cell growth by phosphorylating Gig/Tsc2 and inactivating the Tsc complex. Dephosphorylation of 'Ser-586' by Phlpp triggers apoptosis and suppression of tumor growth. (PubMed:10587646, PubMed:10962553, PubMed:11740943, PubMed:11752451, PubMed:11872800, PubMed:12172554, PubMed:12893776, PubMed:14525946, PubMed:15466161, PubMed:15712201, PubMed:9601646).
Subcellular location: Cytoplasm, cytosol. Cell membrane. Note=Recruited to plasma membrane upon activation.
Tissue specificity: Ubiquitously expressed. Present in ovary, where it is concentrated at the basal side of follicle cells. (PubMed:7876156).
Developmental stage: Expressed both maternally and zygotically. Strongly expressed in embryo and pupa. Weakly expressed in larva. Mildly expressed in adult. (PubMed:15712201, PubMed:7876156).
Domain: Binding of the PH domain to the phosphatidylinositol 3- kinase alpha (PI(3)K) results in its targeting to the plasma membrane.
Ptm: Phosphorylated and activated by Pk61C/PDK1. Phosphorylated on Ser-586 by the rictor-Tor complex. (PubMed:10962553, PubMed:11344272, PubMed:15718470, PubMed:18327897).
Disruption phenotype: Death at the first instar larval stage. (PubMed:9601646).

(Information from UniProt)

akt-2 (WBGene00000103) associated phenotypes

fat content increased, germ cell hypersensitive ionizing radiation, hypersensitivity to mutagen

(Information from WormBase)

akt-2 (UniProt:Q9XTG7) annotation

Function: Acts downstream of age-1 and pdk-1 in the daf-2/insulin receptor-like transduction pathway. Essential role in regulating developmental arrest at the dauer stage. Phosphorylates Forkhead-related daf-16 and the longevity-promoting skn-1 transcription factors, which inhibits their entry into the nucleus and antagonizes their functions. Role in immune function and pathogen resistance. (PubMed:10364160, PubMed:11381260, PubMed:11747825, PubMed:15068796, PubMed:18358814, PubMed:18782349, PubMed:9716402).

Cofactor:Mg(2+)

Enzyme regulation: Phosphorylated and activated by pdk-1. (PubMed:15068796).

Subunit: Interacts with pdk-1, sgk-1, akt-1 and daf-16. Part of a complex containing sgk-1, akt-1 and akt-2. (PubMed:15068796).

Tissue specificity: Expressed in neurons, muscle cells of the pharynx, rectal gland cells, and spermatheca. (PubMed:9716402).

Developmental stage: Expressed in late stage embryos and throughout life. (PubMed:9716402).

Disruption phenotype: Defective egg-laying and increased resistance to pathogens. Simultaneous knockdown of akt-1 and akt-2 result in dauer formation and a weak extension to life span. (PubMed:15068796, PubMed:18782349).

(Information from UniProt)
